# A Review of Fault Diagnosis Technology for Rotating Machinery Based on Deep Generative Models

**DOI:** 10.3390/s26216883

**Published:** 2026-10-30

**Authors:** Guoqiang Pang, Shuyong Liu, Qingchao Yang, Haoyu Zhang

**Affiliations:** Naval Architecture and Marine Engineering, Naval University of Engineering, Wuhan 430032, China; guoqiang_2000@163.com (G.P.); lsydh@sina.com (S.L.); zhanghy139763846@163.com (H.Z.)

**Keywords:** rotating machinery, fault diagnosis, non-stationary operating conditions, few-shot learning

## Abstract

**Highlights:**

**What are the main findings?**

- Impacts of non-stationary conditions and small samples on fault diagnosis are revealed.
- Deep generative models emerge as powerful tools to overcome these data limitations.

**What are the implications of the main findings?**

- Key applications span data augmentation, domain adaptation, and multimodal fusion.
- Critical bottlenecks remain in model interpretability, tuning complexity, and edge deployment.

**Abstract:**

Fault diagnosis of rotating machinery frequently encounters the compounded challenges of non-stationary operating conditions and small-sample data constraints. Traditional deep learning models often exhibit suboptimal performance when addressing these issues. Deep Generative Models (DGMs) offer innovative solutions by synthesizing high-fidelity data samples, reconstructing latent system states, and modeling complex multimodal data streams. This paper presents a review of rotating machinery fault diagnosis technologies based on DGMs. The state-of-the-art applications of DGMs and their variants, including Deep Autoregressive Models (DARs), Variational Autoencoders (VAEs), Generative Adversarial Networks (GANs), Diffusion Models, and Large Language Models (LLMs), are systematically summarized. Specific applications discussed include data generation, domain adaptation and generalization, multimodal data fusion, as well as downstream fault diagnosis and anomaly detection. Furthermore, this review contrasts DGMs with traditional signal processing methods and standard deep learning models. Current limitations are also critically analyzed, focusing on parameter-efficient fine-tuning strategies, interpretability (reliability), and computational efficiency for edge deployment. Finally, future research directions are proposed, emphasizing few-shot or zero-shot learning, robust multimodal data generation, physics-informed hybrid DGM architectures, and the integration of reinforcement learning with DGMs to further enhance diagnostic accuracy in practical scenarios.

## 1. Introduction

Rotating machinery, including electric motors, gearboxes, steam turbines, and propulsion shaft systems, is extensively utilized across various sectors, such as smart manufacturing, marine engineering, vehicle engineering, and aerospace [1,2]. In service, these systems commonly operate under variable conditions, such as speed, load, and temperature fluctuations, and are influenced by complex environmental factors [3]. Non-ideal factors, such as installation deviations, material fatigue damage, and lubrication failure, further exacerbate the risk of mechanical failures [4,5,6]. The operational health of rotating machinery is directly linked to production safety and economic profitability; failure to detect and mitigate early-stage faults may result in severe economic losses or even catastrophic safety accidents [7,8,9]. Consequently, research on fault diagnosis for rotating machinery holds profound engineering significance and practical value.

In practical applications, fault diagnosis of rotating machinery primarily faces the following challenges:

(1) Non-stationary operating conditions, such as speed fluctuations [10], load variations, and background noise [11], complicate the extraction of fault features.
(2) Small-sample dilemmas, including the difficulty of acquiring fault samples [12] and the high cost of data annotation [13], lead to a scarcity of available training data.

Existing studies have predominantly focused on either analyzing signal characteristics under non-stationary conditions or overcoming model training challenges in small-sample scenarios. However, comprehensive investigations addressing the concurrent presence of both challenges remain insufficient.

To systematically review the frontier advancements in the field of rotating machinery fault diagnosis, this paper conducts a bibliometric analysis targeting the dual constraints of non-stationary conditions and small samples. The data sources encompass mainstream academic databases, including Web of Science, Scopus, IEEE Xplore, and Google Scholar, alongside preprint repositories such as arXiv and SSRN. The search covered literature published up to July 2026, including preprints and early-access articles, to capture the most recent findings that had not yet been formally published. The search strategy centered on rotating machinery and associated synonymous terms (e.g., bearings, gears, and rotors), focusing on complex operating environments characterized by non-stationarity, variable working conditions, and intense noise. Keywords and their variants related to small samples, data imbalance, and sample scarcity were also incorporated. Duplicate records are screened and removed. Through this search strategy, which balances high recall and high precision, this paper aims to present a relatively comprehensive and objective overview of the continuously evolving technological trajectory and research landscape in this field.

**Figure 1:**
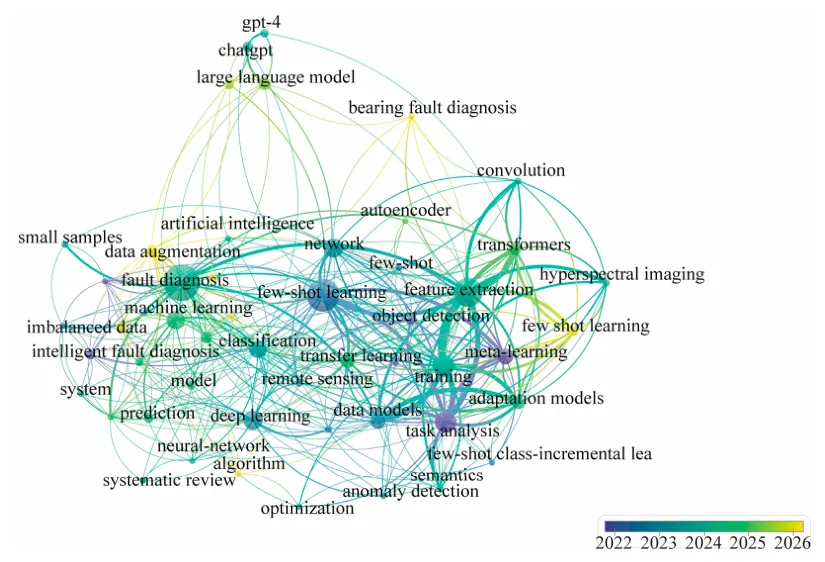
Keyword co-occurrence in fault diagnosis of rotating machinery.

To elucidate the intrinsic correlations and evolutionary trends within the knowledge structure of rotating machinery fault diagnosis, VOSviewer (version 1.6.21) software was employed to construct a keyword co-occurrence network map (Figure 1). It is evident that node clusters centered on fault diagnosis, small-sample learning, and feature extraction occupy the core of the network, exhibiting high connection density and semantic relevance, thereby establishing the current research foundation of this field. Furthermore, temporal overlay visualization reveals a distinct dynamic shift in research hotspots. The focus is gradually transitioning from traditional machine learning frameworks and basic classification tasks toward frontier directions, including Transformers, Large Language Models, and Data Augmentation. This phenomenon indicates that current research is at a critical juncture, transforming from traditional deep learning toward physics-informed fusion and cross-domain knowledge transfer. Although existing literature has separately addressed non-stationary conditions and small-sample dilemmas, comprehensive research targeting their concurrent scenario has not yet formed an independent and cohesive cluster within the network. This highlights a significant research gap in this intersectional domain, necessitating further exploration to resolve the more complex practical challenges encountered in industrial applications.

## 2. Fault Diagnosis Dilemma Under Dual Constraints

### 2.1. Modulation Mechanism of Fault Characteristics Under Non-Stationary Operating Conditions

#### 2.1.1. Frequency Smearing and Amplitude Modulation

Non-stationary operating conditions constitute one of the major physical challenges in rotating machinery fault diagnosis [14]. Under variable speed and load conditions, the intervals of fault-induced impacts vary in real-time with the rotational speed, leading to frequency smearing and the broadening of sidebands in the frequency spectrum. Consequently, conventional Fourier analysis struggles to effectively extract fault characteristic frequencies. Gu et al. [15] demonstrated that vibration signals from wind turbine bearings exhibit strong nonlinearity and non-stationarity, accompanied by severe noise contamination, which makes demodulation difficult for conventional methods. Similarly, Shi et al. [16] discovered that the non-stationarity of rolling bearing vibration signals under variable operating conditions often leads to the failure of traditional signal processing techniques, constituting a major bottleneck in feature extraction. To address the strong non-stationary characteristics of aero-engine vibration signals, Ying et al. [17] proposed an optimized S-transform to enhance time-frequency representation, verifying the decisive impact of non-stationary feature extraction on diagnostic accuracy. Under non-stationary operating conditions, fault vibration signals exhibit joint amplitude, frequency, and phase modulations. This causes the periodic sequence of fault impacts to degenerate into a non-stationary amplitude- and frequency-modulated (AM-FM) process, thereby increasing the difficulty of fault diagnosis. Let the vibration signal $x\left(t\right)$ be modeled in an AM-FM form, as expressed in Equations (1) and (2):

(1) $$x\left(t\right)=A\left(t\right)\cdot \cos\left(2\pi \int_{0}^{t}f_{i}\left(\tau \right)d\tau +\varphi_{0}\right)+n\left(t\right)$$

(2) $$f_{fault}\left(t\right)=O\cdot f_{i}\left(t\right)$$

where $A\left(t\right)$ is the instantaneous amplitude function. Affected by load fluctuations and the decay of fault impacts, $A\left(t\right)$ is proportional to the dynamic excitation force under variable load conditions, leading to the fluctuation of fault impact energy. $f_{i}\left(\tau \right)$ represents the instantaneous rotational frequency at the integration time τ, which is no longer constant under variable speed conditions. $\varphi_{0}$ is the initial phase. $n\left(t\right)$ is the additive noise term. $\int_{0}^{t}f_{i}\left(\tau \right)d\tau$ denotes the total rotational phase from the initial time to the current time t. $f_{fault}\left(t\right)$ is the fault characteristic frequency at time t. O is the fault order ratio. $f_{i}\left(t\right)$ is the instantaneous rotational frequency at time t. This model reveals the fundamental reason for the failure of conventional methods: when $f_{i}\left(t\right)$ varies continuously with time, fault impulses no longer appear as discrete spectral lines in the Fourier spectrum. Instead, they broaden into continuous frequency smearing bands. This causes severe overlap between the fault sidebands and the background noise bands, leading to a drastic degradation in the signal-to-noise ratio (SNR). Although classical order tracking techniques can transform the signal into the angular domain via resampling, accurately estimating the instantaneous phase is highly challenging under conditions of rapid acceleration and deceleration. Consequently, the resulting order tracking errors prevent the resampled fault features from being effectively focused.

#### 2.1.2. Distortion Effect

Non-stationary operating conditions not only modulate the fault source signal itself but also induce complex distortions on fault impact pulses through time-varying transfer paths. In planetary gearboxes, the revolution of planet gears leads to periodic amplitude modulation of meshing vibrations. The fault characteristics are masked by the “through effect”, resulting in severe aliasing between the fault impact components and structural vibration components in the time-frequency domain. Yu et al. [18] pointed out that for planetary bearing vibration signals, the time-varying transfer path causes the transmission route from the fault to the accelerometer to vary periodically with the rotation of the planet carrier, leading to complex non-stationary and nonlinear characteristics in the signals. Sun et al. [19] further revealed in the non-contact diagnosis of gearboxes that acoustic and thermal features under different fault conditions are also significantly affected by transfer path modulation. Similarly, Lv et al. [20] observed in the fault diagnosis of railway vehicle bogies that the inadequate representation of fault features under strong noise backgrounds is fundamentally attributed to transfer path distortion and environmental interference. The distortion effect of the time-varying transfer path on the impact pulse can be described as a variable-path convolution between the sensor-acquired signal $y\left(t\right)$ and the fault source signal $s\left(t\right)$, as expressed in Equation (3):

(3) $$y\left(t\right)=\int_{-\infty }^{\infty }h\left(t,\tau \right)\cdot s\left(\tau \right)d\tau +e\left(t\right)$$

where $s\left(t\right)$ represents the periodic impact sequence generated by the fault source (e.g., inner race spalling impacts in bearings). $h\left(t,\tau \right)$ is the time-varying impulse response function, denoting the response at time t to an impact occurring at the delayed time τ. During rotor run-up or load variations, parameters such as bearing clearance, contact angle, and housing resonance modes change, leading to non-stationary variations in the amplitude-frequency characteristics of the transfer path. $e\left(t\right)$ denotes the background noise.

Direct time-domain analysis of the time-varying impulse response $h\left(t,\tau \right)$ fails to intuitively reveal its frequency-selective characteristics. To address this, the time-varying frequency response function $H\left(\omega ,t\right)$ is introduced as its equivalent description in the frequency domain. The Fourier transform of $h\left(t,\tau \right)$ is performed along the delay axis τ at a frozen time instant t, as expressed in Equation (4):

(4) $$H\left(\omega ,t\right)=\int_{-\infty }^{\infty }h\left(t,\tau \right)e^{-j\omega \tau }d\tau$$

where ω is the angular frequency, j is the imaginary unit, and τ is the integration variable representing the historical time instant of the fault impact occurrence. t denotes the current observation time. $H\left(\omega ,t\right)$ characterizes the complex gain of different angular frequency components ω passing through the transfer path at time t. Furthermore, taking the modulus of $H\left(\omega ,t\right)$ yields the instantaneous amplitude-frequency gain, $\left|H\left(\omega ,t\right)\right|$, which can be used to evaluate the degree of distortion of different frequency components under the time-varying path.

#### 2.1.3. Feature Distribution Shift

Another challenge introduced by non-stationary operating conditions is feature distribution shift. Vibration signals collected under varying rotational speeds and load ranges exhibit significant differences in data distribution. This inherent domain shift leads to the performance degradation of models trained under constant operating conditions when applied to variable speed scenarios. Li et al. [21] revealed significant differences in the data distribution of rolling bearings under various operating conditions through shapelets time-series analysis. Zhai et al. [22] observed a synergistic deterioration effect between the difficulty of feature extraction caused by signal non-stationarity and the low diagnostic accuracy under small-sample conditions. Cao et al. [23] analyzed the degree of distribution shift between the source domain and the target domain under variable operating conditions, pointing out that inter-domain discrepancy is the most critical technical bottleneck in transfer diagnosis. Zhong et al. [24] further quantified the domain distribution differences caused by variable operating conditions in train bearing diagnosis, finding that complex and variable operating conditions lead to a nonlinear increase in the distribution distance between the source and target domains. To quantify the degree of feature distribution shift in non-stationary environments, the Kullback–Leibler (KL) divergence is adopted to measure the distribution discrepancy before and after the operating condition changes. Let the source domain feature distribution before the operating condition change be $P_{source}\left(x\right)$, and the target domain distribution after the change be $P_{target}\left(x\right)$, where x is a univariate feature random variable extracted from the vibration signal (e.g., frequency band energy, time-domain statistics). The KL divergence between these two distributions is defined as Equation (5):

(5) $$D_{KL}\left(P_{source}∥P_{target}\right)=\int P_{source}\left(x\right)\log\frac{P_{source}\left(x\right)}{P_{target}\left(x\right)}dx$$

This metric measures the amount of information lost when the target domain distribution $P_{target}\left(x\right)$ is used to approximate the source domain distribution $P_{source}\left(x\right)$. A larger value indicates a more severe feature distribution shift caused by operating condition variations.

When the features are assumed to follow a Gaussian distribution, let the mean and covariance matrix of the source domain features be $\mu_{s}$ and $\Sigma_{s}$, and those of the target domain be $\mu_{t}$ and $\Sigma_{t}$, respectively. The degree of distribution shift between the two domains can then be quantified using the Mahalanobis distance. If it is further assumed that the covariance structures of the two distributions are identical (i.e., $\Sigma_{s}$ = $\Sigma_{t}$ = Σ), the Mahalanobis distance can be simplified as Equation (6). By normalizing and weighting each feature dimension through the inverse of the covariance matrix, the Mahalanobis distance eliminates the effects of measurement units and inter-variable correlations, thereby more accurately reflecting the substantial shift of the distribution center in the feature space.

(6) $$D_{M}=\sqrt{{\left(\mu_{s}-\mu_{t}\right)}^{T}\Sigma^{-1}\left(\mu_{s}-\mu_{t}\right)}$$

However, the distribution shift under variable speed conditions is non-ergodic, causing the feature subspace learned under constant operating conditions to suffer severe distortion when applied to variable conditions. More challengingly, even if the marginal distribution $P\left(x\right)$ is aligned through domain adaptation techniques, the conditional distribution $P\left(y|x\right)$ (i.e., the spectral morphology distribution of features x corresponding to the same fault category y under varying speeds) still undergoes drastic variations. This makes the compensation for feature distribution shift far more complex than simple distribution alignment. Addressing the distribution shift problem requires models with powerful representation capabilities, which typically rely on massive amounts of data. However, the practical reality is characterized by severe data scarcity.

### 2.2. Challenges in Statistical Learning Under Small Sample Constraints

#### 2.2.1. Curse of Dimensionality and Manifold Destruction

Small sample constraints violate the prerequisite of deep learning, which relies on massive data to cover the underlying manifold, thereby subjecting fault diagnosis to the curse of dimensionality and manifold destruction [25]. In high-dimensional feature spaces, a limited number of samples leads to extreme sparsity in data distribution, causing the originally continuous and compact fault data manifold in low-dimensional space to fracture. This loss of geometric continuity makes it difficult for models to reconstruct the complete fault distribution from discrete samples, thereby failing to establish a robust feature mapping. Li et al. [26] revealed that when label information is scarce, the fundamental cause of the performance degradation in supervised learning is precisely the inability of limited samples to adequately represent the manifold structure in high-dimensional space. Yu et al. [27] noted that fault signals from large-scale rotating machinery are not only superimposed with strong noise but also suffer from a scarcity of effective samples, making it difficult to extract valid inter-sample correlations.

#### 2.2.2. Overfitting and Shrinking Generalization Bound

The dual dilemma caused by sparse annotations is further exacerbated: on the one hand, models are prone to overfitting to the noise patterns of limited training samples rather than the true fault mechanisms; on the other hand, the shrinking generalization bound leads to degraded performance under unseen operating conditions. Chang et al. [28] investigated a meta-learning network designed for small-sample prior conditions, pointing out that the scarcity of labeled data is a critical obstacle hindering the widespread application of intelligent fault diagnosis methods. Sun et al. [29] found in automotive transmission diagnosis that traditional deep learning methods (e.g., PLATIPUS and CNN) achieved an accuracy of only 87.5% in 1-shot learning scenarios, which is a direct manifestation of overfitting and generalization bound shrinking. Chai et al. [30] further demonstrated that deep learning methods relying on large-scale labeled datasets are highly susceptible to overfitting in cross-condition scenarios, thereby limiting their generalization capabilities. To address the dilemmas of rotating machinery fault diagnosis caused by non-stationary operating conditions and small sample constraints discussed in Section 2.1 and Section 2.2, a straightforward solution is to augment the sample size for each operating condition through data augmentation [31], or to introduce regularization constraints via probabilistic models to suppress overfitting [32]. In this direction, generative models have attracted considerable attention due to their ability to simulate data distribution characteristics.

### 2.3. Limitations of Classical Generative Models and the Rise of Deep Generative Models

When confronting the dilemmas of non-stationary operating conditions and small sample constraints, the limitations of classical generative models (e.g., GMM and ARIMA) primarily stem from their shallow architectures, limited representation capabilities, and lack of prior knowledge [33,34]. These models are typically built upon assumptions such as stationarity or Markovian properties. When the data distribution drifts over time or highly nonlinear couplings exist among variables, their predefined statistical assumptions are violated, making them incapable of accurately capturing dynamic evolutionary patterns. Under conditions of data scarcity, due to the lack of knowledge transfer capabilities derived from pre-training, these models are highly susceptible to overfitting the noise present in the training data [35]. Furthermore, they struggle to accurately estimate the joint probability distribution from scratch, resulting in poor generalization performance. When the coupled effects of non-stationarity and small sample sizes occur simultaneously, the models are required to adapt to dynamic changes while lacking sufficient data support, which often leads to suboptimal performance and an inability to provide effective support for downstream tasks. Consequently, researchers have gradually shifted their focus toward DGMs [36].

## 3. Solution Paradigms of Deep Generative Models

### 3.1. Principles of Deep Generative Models

**Figure 2:**
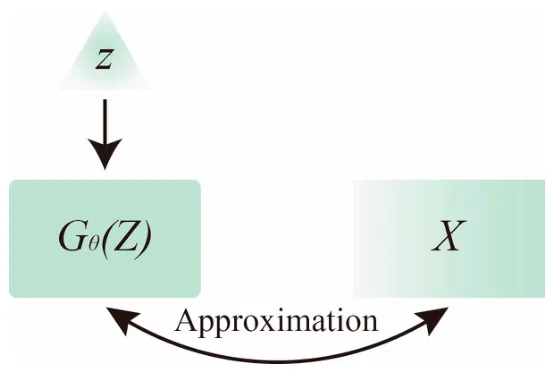
Schematic diagram of the deep generative model.

From a mathematical perspective, the core objective of Deep Generative Models (DGMs) is to learn a representation of an intractable probability distribution X. This distribution is defined in the $R^{m}$ space, where the dimension m is typically high. Complex characteristics are exhibited by the distribution. This learning process relies on a large set of independent and identically distributed (i.i.d.) samples. These samples are drawn from X and constitute the training data. Explicit probability expressions are sought by traditional statistical inference. In contrast, the primary purpose of DGMs is to construct a generator G. This is expressed in Equation (7) [37]:

(7) $$G:R^{k}\to R^{m}$$

where the generator G serves to map data from a tractable distribution Z in the $R^{k}$ space to data that approximates the target distribution X. As illustrated in Figure 2, this implies that for every sample x drawn from X, there exists at least one latent vector z∼Z satisfying G(z)≈x. Consequently, the transformed distribution G(z) constitutes an approximation of X.

### 3.2. Deep Generative Models and Their Variants

#### 3.2.1. Deep Autoregressive Models

As a branch of DGMs, the core mechanism of Deep Autoregressive Models (DARs) lies in synthesizing new samples through iterative inference by utilizing past observational data [38,39]. This mechanism endows the model with distinct advantages in sequential data modeling tasks, such as time-series forecasting. Assume a dataset $D={\left\{x_{i}\right\}}_{i=1}^{n}$ containing n samples, where x is an m-dimensional real-valued vector. The parameter optimization process of the DAR model aims to minimize the negative log-likelihood of the data fitting via the probability chain rule [40], as expressed in Equation (8):

(8) $$L_{DAR}=-E_{x∼p_{data}}\left[\log p_{model}\left(x_{i}|x_{1},\ldots x_{i-1};\theta \right)\right]$$

where $p_{model}\left(x_{i}|x_{1},\ldots x_{i-1};\theta \right)$ denotes the conditional probability density function parameterized by θ, which characterizes the probability distribution of the current observation $x_{i}$ conditioned on the historical sequence $x_{1},\ldots ,x_{i-1}$. The operator E represents the expectation over the data distribution. Fundamentally, the training objective is to enable the model to capture temporal dependencies from historical contexts, thereby maximizing the likelihood of subsequent observations within the sequence.

#### 3.2.2. Variational Autoencoders

Variational Autoencoders (VAEs) represent a typical category of DGMs. Data compression and reconstruction are focused on [41]. A dual-network architecture is comprised by the model. An encoder and a decoder are included. High-dimensional observational data are mapped into a low-dimensional latent space by the former. Key features are thereby extracted. The original input is reconstructed by the latter based on these latent representations. Dimensionality reduction is essentially achieved by this mechanism. Latent variables that capture the intrinsic structure of the encoded data are exploited. Mathematically, the VAE is formulated as a probabilistic model. Latent variables $z\in R^{k}$ are introduced. A specific prior distribution (e.g., a standard normal distribution) is followed. The input vector x∈X is thereby processed. A dataset $D={\left\{x_{i}\right\}}_{i=1}^{n}$ containing n samples is considered, where $x_{i}\in R^{m}$. The training objective of the model is to optimize the data log-likelihood during the generation process. This is expressed in Equation (9):

(9) $$\log p_{\theta }\left(x_{1},\ldots x_{n}\right)=\log\prod_{i=1}^{n}p_{\theta }\left(x_{i}\right)$$

To effectively maximize this log-likelihood, VAEs optimize the Evidence Lower Bound (ELBO) for each data point $x_{i}$, which is defined as Equation (10) [42]:

(10) $$\begin{array}{ll} {\log p}_{\theta }\left(x_{i}\right) & =\log\int p_{\theta }\left(x_{i}|z\right)p\left(z\right)\frac{q\left(z|x_{i}\right)}{q\left(z|x_{i}\right)}dz\\ & ={\log E}_{z∼q_{\phi }\left(z|x_{i}\right)}\left[p_{\theta }\left(x_{i}|z\right)\frac{p\left(z\right)}{q\left(z|x_{i}\right)}\right]\\ & \geq \underset{\mathrm{reconstruction}\;\mathrm{error}}{\underbrace{E_{q_{\phi }\left(z|x_{i}\right)}\left[{\log p}_{\theta }\left(x_{i}|z\right)\right]}}-\underset{\mathrm{regularisation}}{\underbrace{D_{KL}\left(q_{\phi }\left(z|x_{i}\right)∥p\left(z\right)\right)}}\\ & =L_{\mathrm{VAE}}\left(x_{i},\theta ,\phi \right) \end{array}$$

where θ represents the parameters of the decoder $p_{\theta }\left(x|z\right)$, which is responsible for reconstructing the data from the latent variables. ϕ denotes the parameters of the encoder $q_{\phi }\left(z|x\right)$, which is utilized to approximate the posterior distribution of the latent variables. $D_{KL}$ represents the KL divergence. By balancing the reconstruction accuracy and the regularization constraints in the latent space, VAEs are capable of not only learning efficient compressed representations but also ensuring that the samples generated from the latent space possess high coherence.

#### 3.2.3. Generative Adversarial Networks

Generative Adversarial Networks (GANs) [43] belong to a class of DGMs whose core lies in adopting a unique adversarial training mechanism. This architecture comprises two competing sub-networks, namely the generator G and the discriminator D. The task of the generator is to synthesize samples (e.g., thermal imaging or vibration acceleration signals) based on random noise, while the discriminator is dedicated to distinguishing whether these generated samples are real data or fabricated ones [44]. Through this adversarial game, the generator can continuously optimize its generation strategy, thereby progressively improving the quality of the output samples and ultimately generating high-fidelity data. The objective function describing this optimization process is formulated as a minimax optimization problem [45], which is expressed as Equation (11):

(11) $$\begin{array}{ll} \underset{\theta_{C}}{\min}\underset{\theta_{D}}{\;\max}L_{GAN} & =\underset{\theta_{C}}{\min}\underset{\theta_{D}}{\;\max}{\;E}_{x∼p_{data}}\left[\log D\left(x;\theta_{D}\right)\right]\\ & +E_{z∼p_{model}}\left[\log\left(1-D\left(G\left(z;\theta_{G}\right);\theta_{D}\right)\right)\right] \end{array}$$

where $L_{GAN}$ denotes the adversarial loss of the GAN, and x represents the data sampled from the data distribution pdata. $p_{model}$ is the probability distribution in the latent space, which is typically assumed to follow a Gaussian distribution.

#### 3.2.4. Diffusion Models

Denoising Diffusion Probabilistic Models (DDPMs) are also known as diffusion models. They have emerged as a fundamental cornerstone of modern generative artificial intelligence [46]. The core of DDPMs lies in the establishment of a forward diffusion process and a reverse denoising process. During the forward diffusion phase, Gaussian noise is gradually injected into the initial data sample $x_{0}$ over T time steps through a predefined variance schedule strategy. The sample is ultimately degraded into an isotropic standard normal noise sample $x_{T}$. Conversely, the reverse denoising process is performed iteratively. This transformation is reversed. The original data distribution is thereby reconstructed from pure noise. Formally, the forward process is defined as a Markov chain ${\left\{x_{t}\right\}}_{t=0}^{T}$. The chain is governed by the forward transition probability $q\left(\cdot \right)$ [46,47]. This is expressed in Equation (12):

(12) $$q\left(x_{t}|x_{t-1}\right)=N\left(x_{t};\sqrt{1-\beta_{t}}x_{t-1},\beta_{t}I\right)\;q\left(x_{1:T}|x_{0}\right)=\prod_{t=1}^{T}q\left(x_{t}|x_{t-1}\right)$$

where $\beta_{t}\in \left(0,1\right)$ denotes the time-dependent noise scale at step t, I is the identity matrix, and N represents the Gaussian distribution. The reverse denoising process, denoted as $p_{\theta }\left(\cdot \right)$, iteratively reconstructs the original data distribution $q\left(x_{0}\right)$ from pure noise $x_{T}$.

Specifically, the reverse process defines another Markov chain governed by [46], as expressed in Equation (13):

(13) $$p_{\theta }\left(x_{t-1}|x_{t}\right)=N\left(x_{t-1};\mu_{\theta }\left(x_{t},t\right),\sum_{\theta }\left(x_{t},t\right)\right)\;p_{\theta }\left(x_{0:T}\right)=p_{\theta }\left(x_{T}\right)\prod_{t=1}^{T}p_{\theta }\left(x_{t-1}|x_{t}\right)$$

where the mean $\mu_{\theta }$ and the covariance $\Sigma_{\theta }$ are learned by a neural network (typically employing a U-Net architecture) and parameterized by θ.

The training loss function aims to maximize the likelihood $p_{\theta }\left(x_{0}\right)=\int p_{\theta }\left(x_{0:T}\right){dx}_{1:T}$ by maximizing the Evidence Lower Bound (ELBO), i.e., $L_{Diff}\leq \log p_{\theta }\left(x_{0}\right)$. The ELBO can be formulated by matching the true denoising distribution $q\left(x_{t-1}|x_{t}\right)$ with the parameterized denoising model $p_{\theta }\left(x_{t-1}|x_{t}\right)$ via [48,49], as expressed in Equation (14):

(14) $$L_{Diff}=-\sum_{t\geq 1}E_{q\left(x_{t}\right)}\left[D_{KL}\left(q\left(x_{t-1}|x_{t}\right)||p_{\theta }\left(x_{t-1}|x_{t}\right)\right)\right]+C$$

where C is a constant. The forward diffusion and reverse denoising processes, as well as the key characteristics of diffusion models, are summarized in Table 1.

#### 3.2.5. Large Language Models

Large Language Models (LLMs) have emerged as a mainstream paradigm for addressing various engineering challenges. These models demonstrate remarkable capabilities in handling diverse inputs, such as text prompts, questions, and visual images, through prompt engineering. Although fundamentally belonging to autoregressive generative models, typical LLMs represented by GPT-4, Llama 4, Claude, and DeepSeek are all based on the Transformer architecture and have been pre-trained on large-scale datasets. Accordingly, they are discussed separately in a dedicated section. By integrating attention mechanisms, this architecture extracts both local and global dependencies among tokens [49]. From a mathematical perspective, LLMs operate as autoregressive models, with the core mechanism being the prediction of the next token based on historical tokens. This process can be formulated via the following conditional probability Equation (15):

(15) $$p(x_{n}|x_{1:n-1})=softmax(Wh_{n-1})$$

where $p(x_{n}|x_{1:n-1})$ denotes the transition probability of the n-th token given the preceding sequence of n−1 tokens, $x_{1:n-1}$. W represents the learnable weight matrix, and $h_{n-1}$ is the hidden state generated by the Transformer after processing the aforementioned token sequence. The softmax function is an activation function that converts logits into a probability distribution for token selection. This implies that after being processed by the softmax function, the model outputs the token with the highest probability.

Table 1 compares the architectures and specific characteristics of the proposed DGMs.

### 3.3. Analysis of Application Scenarios

**Figure 3:**
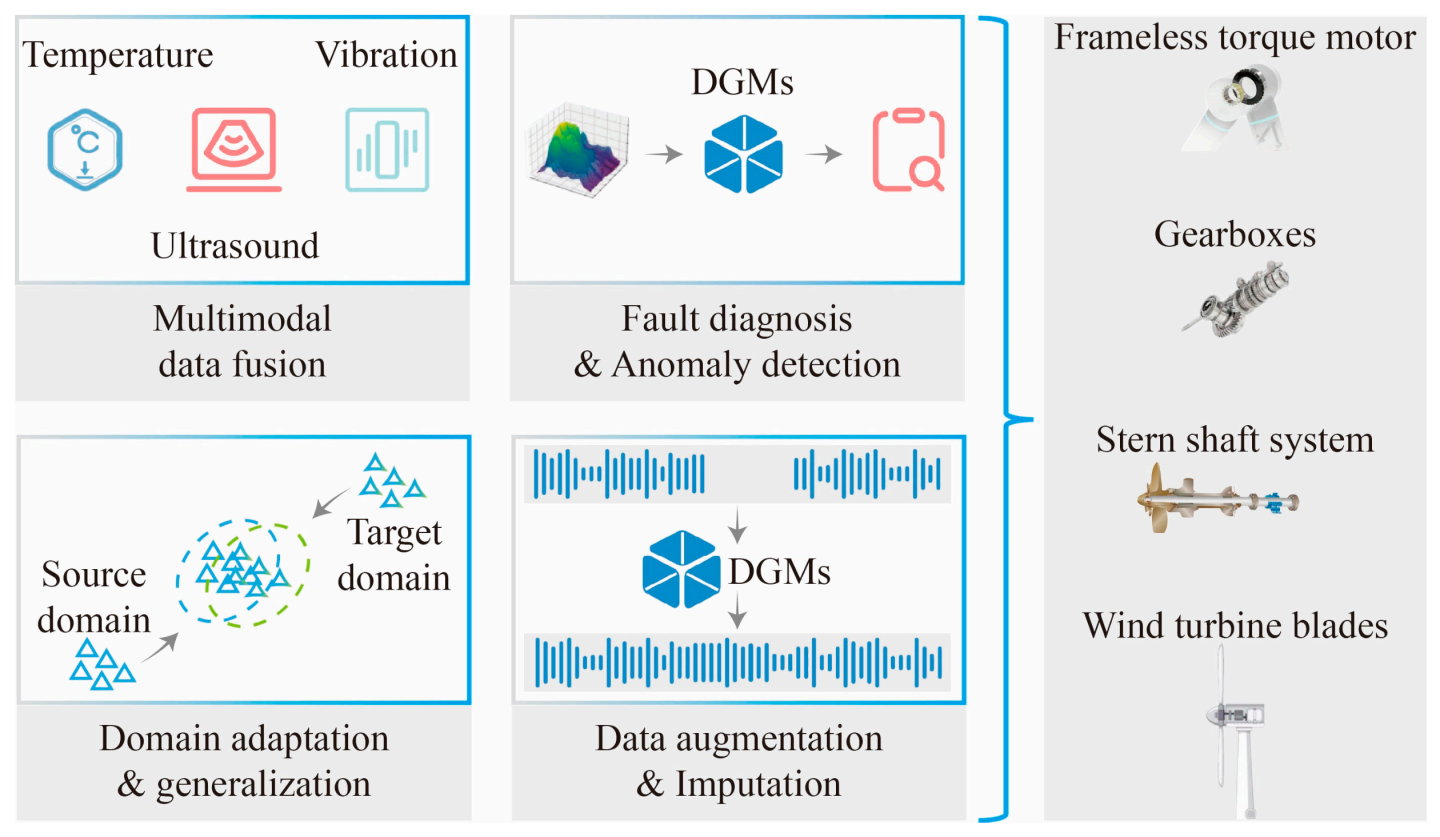
The Application of Deep Generative Models in Fault Diagnosis of Rotating Machinery.

The preceding section provides a brief overview of state-of-the-art DGMs. As illustrated in Figure 3, this section explores the specific applications of DGMs in rotating machinery fault diagnosis systems, particularly across various task scenarios.

#### 3.3.1. Performance Metrics

Table 2 lists some of the metrics used to evaluate the performance of DGMs, which have been widely applied across various downstream tasks. Taking Mean Absolute Error (MAE) as an example, it not only measures the agreement between synthetic and real data in data augmentation tasks but also serves as an evaluation criterion for fault diagnosis and anomaly detection (FD & AD) tasks. Furthermore, the Pearson Correlation Coefficient (PCC) is specifically utilized to quantify the similarity characteristics between real and synthetic data in augmentation tasks.

#### 3.3.2. For Data Generation

**Figure 4:**
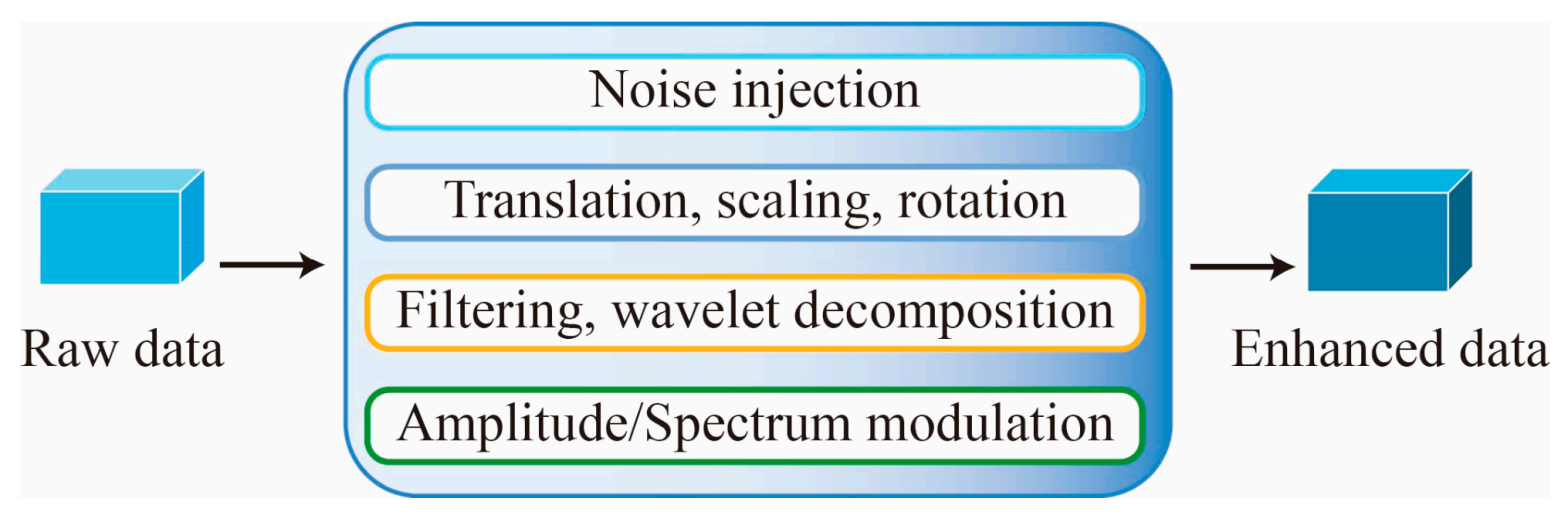
Data Transformation-Based Augmentation Methods.

As a macroscopic concept, data generation encompasses techniques such as data augmentation, reconstruction, and imputation, with their core differences lying in the generation purposes and underlying mechanisms. Specifically, data augmentation strengthens model robustness by deriving new samples; data reconstruction is dedicated to restoring signals from incomplete or compressed information; and data imputation focuses on repairing missing or corrupted data to refine datasets. According to the classification system in [55], these techniques are primarily categorized into three types: Synthetic Minority Over-sampling Technique (SMOTE), data transformation-based augmentation methods, and generative model-based methods. SMOTE aims to address class imbalance issues by synthesizing minority class samples. Data transformation-based augmentation methods enrich dataset diversity by means of noise injection or image manipulation. Generative model-based methods, specifically referring to the DGM approaches in this paper, are also included. Figure 4 shows the enhanced methods of one-dimensional time series in fault diagnosis applications.

Data augmentation methods based on DGMs have become indispensable tools for addressing challenges such as severe noise and data scarcity in practical industrial scenarios. By synthesizing high-quality data that reflects the statistical characteristics of real data samples, these models achieve robust supplementation and enhancement of existing datasets. This approach is particularly crucial for acquiring experimental data that is costly and labor-intensive to collect [56,57]. Therefore, by adopting DGM-based data augmentation techniques, the industrial sector is expected to alleviate data scarcity and resolve the inherent class imbalance problems in industrial environments. This expands the training data for downstream FD & AD tasks while maintaining the fidelity of the original data distribution [58,59].

Beyond data augmentation, DGMs also play a vital role in data imputation, providing effective solutions for the missing data problem in industrial systems [60]. Data missingness can severely impair the diagnostic or detection performance of models [61]. The inherent capability of DGMs to learn complex data distributions makes them highly suitable for data imputation tasks. Rather than simply filling missing values with fixed estimates, DGMs can leverage the latent patterns and correlations within the data to generate plausible and credible values for the missing entries.

Existing literature on the application of DGMs to data augmentation and imputation tasks is summarized in Table 3. Given the differences in datasets, operating conditions, tasks, and evaluation metrics, the results presented below should be interpreted as reference comparisons only. Utilizing the performance metrics outlined in Table 2, signal processing methods, traditional machine learning (ML) methods (such as SMOTE, Recurrent Neural Networks (RNN), Long Short-Term Memory (LSTM) networks, and Gated Recurrent Units (GRU)), and DGM-based methods are quantitatively evaluated. SMOTE-based methods are classified as traditional ML, as they produce synthetic data through linear interpolation between minority samples and their k-nearest neighbors (KNN) [62].

Three main conclusions can be drawn from Table 3. First, most studies are confined to a single data augmentation or imputation task, lacking cross-method comparative analyses. Furthermore, in terms of evaluation, DDPM and GAN models generally achieve better performance than traditional machine learning methods, such as LSTM and SMOTE, on metrics including MMD and EMD. Finally, there remains a scarcity of cross-comparative studies among signal processing, traditional deep neural networks, and DGMs. This is largely because signal processing techniques are usually limited to preprocessing, DGMs are often regarded as auxiliary data-generation tools, and physical knowledge is not explicitly integrated into these models. Furthermore, diffusion-based models may involve architectures with billions of parameters. In practice, training typical diffusion models can require more than 10 h, even on commercial-grade GPUs. Recent studies suggest that the number of diffusion steps is a key factor in generation quality, typically ranging from 500 to 1000 steps [92,148,149].

#### 3.3.3. For Domain Adaptation and Generalization

**Figure 5:**
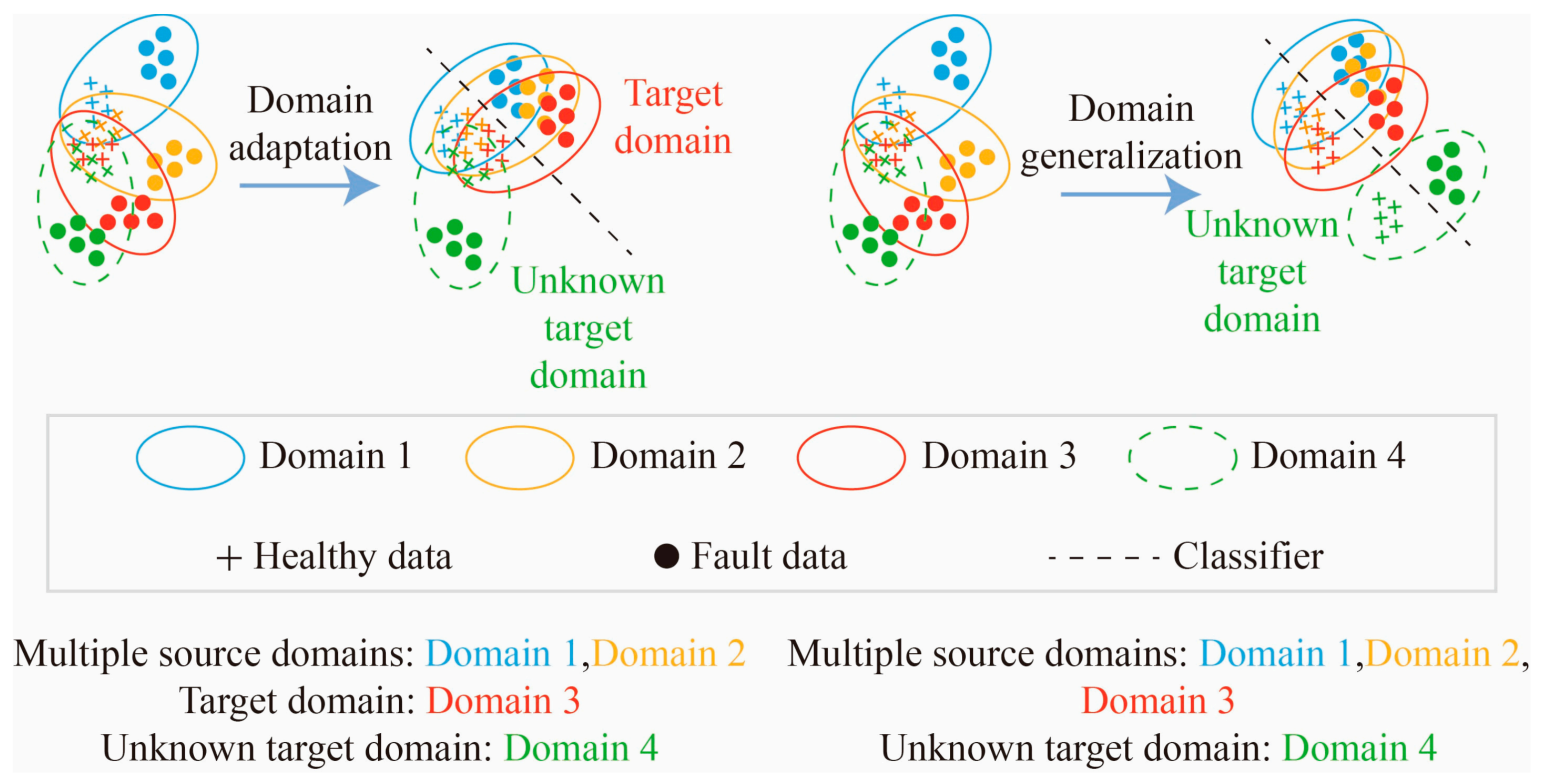
Schematic diagram of domain adaptation and domain generalization. Domain adaptation minimizes the distribution gap between source and target domains, whereas domain generalization transfers knowledge to an unseen target without accessing its data.

Transfer learning (TL) transfers knowledge learned in a source task to enhance performance on related target domains or tasks [150,151]. Domain Adaptation (DA) and Domain Generalization (DG) are two key directions in this framework. Domain adaptation reduces the distribution discrepancy between source and target domains [152,153], learns transferable representations from multiple source domains to generalize to unseen target domains without using target-domain data during training. Their main difference lies in whether target-domain data are accessible during training. Domain generalization is therefore more suitable for evaluating model robustness when target-domain data are unavailable [83], as illustrated in Figure 5.

The applications of DGMs within the TL framework for domain adaptation and domain generalization are summarized in Table 3. Most existing studies are concentrated on GAN-based domain adaptation models, including:

(1) translation from damaged to undamaged data under scarce damage data [131,132,133];
(2) adaptation between simulated and experimental data [134,137,141];
(3) adaptation to different types of rotating machinery, rotational speeds, or operating conditions [88,135,136].

A limited number of studies [91,144,145,146,147] have addressed applications under unknown or unseen operating conditions. Meta-learning [146,147] has been employed as a complementary approach, in which models are trained to leverage knowledge acquired from multiple related tasks for rapid adaptation to new tasks or environments. This is particularly relevant because many deep generative model-based domain generalization methods rely primarily on statistical learning from available data, without explicit incorporation of system physics. Given the high demand for domain generalization in industrial scenarios where target-domain data are unavailable, an emerging trend has been observed in applying diffusion models to domain generalization for rotating machinery fault diagnosis systems [105]. It should be noted that certain studies cover multiple application categories. For example, methodologies combining data augmentation with domain generalization have been proposed in [88,91,105] to address unseen target domains, as shown in Table 3. These methods inherently span both categories, as both augmentation of scarce data and generalization to unseen domains are achieved.

#### 3.3.4. For Multimodal Data Fusion

The application of DGMs in multimodal data fusion for condition monitoring remains in its early stages. Notably, DGMs based on diffusion mechanisms and large language models (LLMs) exhibit unique development potential. This is largely attributed to the widespread adoption of the Transformer architecture, which maps heterogeneous data, including text, visual images, and audio inputs, into a shared latent space. The attention mechanism within the Transformer framework [49] provides a solid foundation for multimodal fusion. Through dynamic computation of attention scores across modalities, input features are weighted in a context-aware manner, enabling information with higher significance or relevance to be prioritized during the fusion process. These capabilities are particularly critical for diffusion- and LLM-based DGMs, as the attention mechanism not only enhances model expressiveness but also ensures consistency between the outputs and the joint distribution.

**Figure 6:**
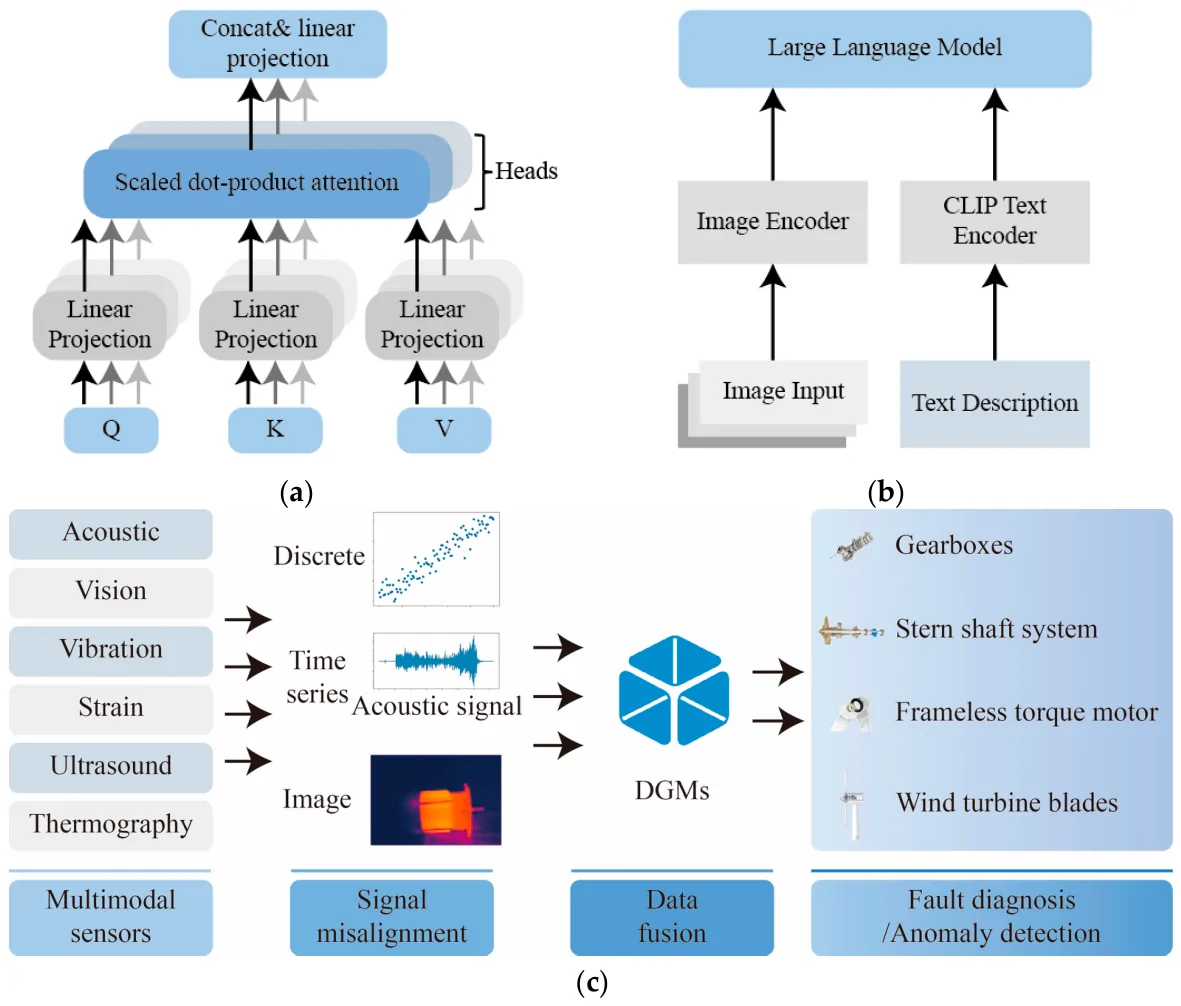
Applications of DGMs in multimodal data fusion. (**a**) Multihead attention mechanism in Transformer-based networks; (**b**) LLM-based image and text prompt modal fusion; (**c**) DGMs apply multimodal data fusion in condition monitoring.

Figure 6a shows the multi-head attention mechanism in Transformer networks. This mechanism is a basic component of diffusion models, such as Stable Diffusion [154], and of large language model (LLM) architectures. Text descriptions are encoded into value (V) matrices by a text encoder, such as the CLIP text encoder [155]. Attention scores are then computed to enable dynamic fusion between image and text modalities.

Figure 6b illustrates cross-modal fusion in LLMs. Paired image and text inputs are processed jointly. Image inputs are passed through an image encoder to a feature extraction module. Latent representations are obtained and projected into key (K) and query (Q) matrices.

Figure 6c shows the main workflow of multimodal data fusion based on deep generative models (DGMs). Multimodal heterogeneous sensors, such as vibration accelerometers, stereo cameras, and acoustic emission sensors, provide discrete data, continuous time series, and images. These data are processed and dynamically fused by DGMs. The fused representations are used to support downstream fault diagnosis and anomaly detection (FD&AD) tasks. Table 4 summarizes the existing literature on the application of DGMs to multimodal data fusion. Given the differences in datasets, operating conditions, tasks, and evaluation metrics, the following results should be interpreted as reference comparisons only.

Table 4 summarizes the applications of DGMs in multimodal data fusion. Diffusion- and LLM-based Transformer models are classified as DGMs, whereas other Transformer architectures are categorized as DNNs. In the field of multimodal data fusion, diffusion- and LLM-based models constitute a significant portion of DGMs. Conversely, studies utilizing Kalman filtering for multimodal fusion are categorized as signal processing methods. Although Kalman filtering is a prevalent and effective approach for real-time multimodal fusion in monitoring scenarios, it exhibits suboptimal performance when handling massive heterogeneous data streams. This limitation indicates that diffusion models and LLM-based models possess broad application potential in future multimodal data fusion technologies.

#### 3.3.5. For Fault Diagnosis and Anomaly Detection

For downstream FD & AD tasks, LLM-based methods and generative model-based methods (such as VAEs, GANs, or diffusion models) play different roles. Within the VAE or GAN frameworks, DGMs are typically employed for signal-level tasks, such as data generation, feature extraction, or anomaly detection based on reconstruction errors. These models primarily operate on the statistical distributions of sensor signals. In contrast, LLM-based methods are not typically used as sensor-level signal generators; rather, they are employed as pre-trained representation learning and reasoning modules that are adapted to diagnostic tasks through fine-tuning. The capabilities of LLMs are largely attributed to large-scale pre-training, through which language understanding, contextual reasoning, and knowledge integration can be leveraged for diagnostic inference. In addition, some multimodal foundation models or modality-extended LLMs can integrate text, images, and sensor signals for cross-modal diagnosis and decision support.

**Figure 7:**
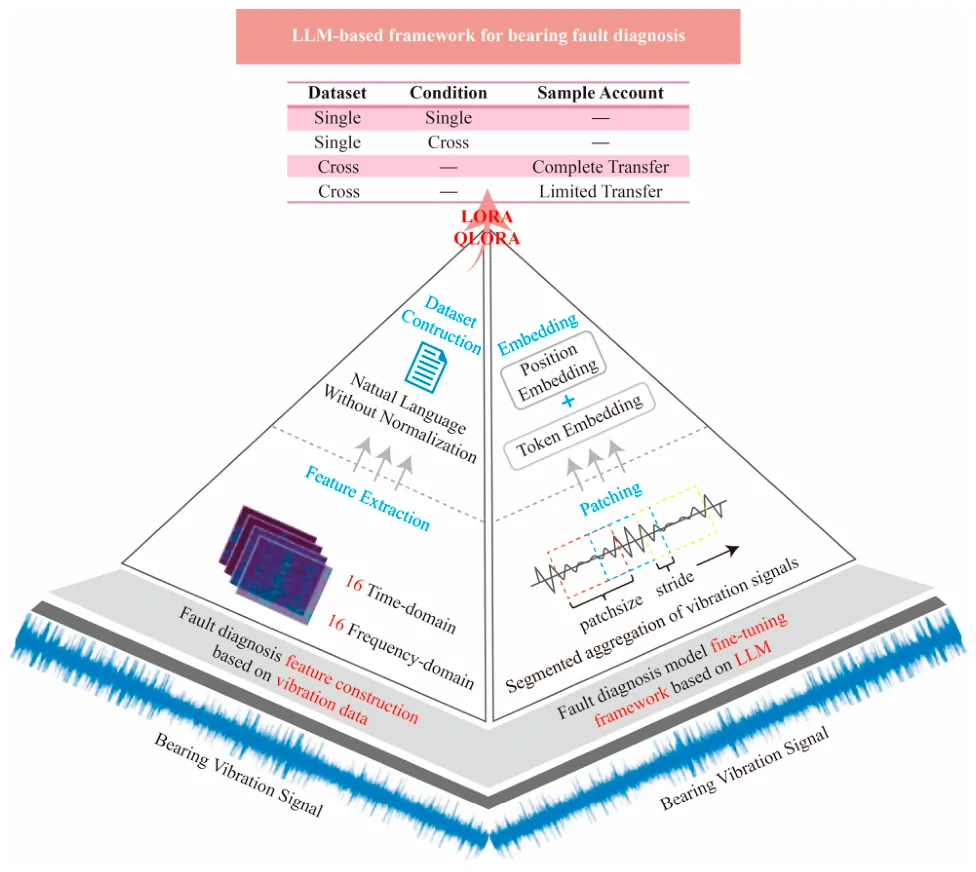
Large language models for bearing fault diagnosis. The fundamental premise of applying LLMs to bearing fault diagnosis is to align vibration signals with textual descriptions, and subsequently fine-tune the models for direct fault diagnosis.

When applying LLMs to FD & AD tasks, their applicability should be distinguished according to model type. Pure language models primarily process textual inputs, such as equipment maintenance records, fault descriptions, diagnostic rules, or natural language queries. To handle sensor signals such as vibration, acoustic, or image data, it is typically necessary to introduce signal encoders and modality alignment mechanisms, or to adopt multimodal foundation models. As illustrated in Figure 7, a GPT-2 model was fine-tuned using Low-Rank Adaptation (LoRA) on a bearing vibration dataset to conduct fault diagnosis research [165]. In Table 4, LLM-based FD & AD tasks targeting wind power systems, bearings, and gearboxes are summarized. Most studies on bearings and gearboxes also adopt fine-tuning techniques such as LoRA [36].

The performance of LLMs in FD and AD tasks depends on fine-tuning strategies and model scale. Zheng et al. [178] compared the diagnostic accuracy of Llama-2 models with different sizes, including 7B and 13B parameters. A 7B-parameter model was found to achieve satisfactory performance in certain scenarios. Larger models were observed to be more suitable for tasks requiring higher accuracy or involving more complex data distributions. Further details on the effects of fine-tuning techniques, fine-tuning strategies, and model scales are provided in Section 4.1.1 and References [179,180,181,182].

## 4. Challenges and Future Perspectives

This subsection summarizes the existing limitations of DGMs in rotating machinery fault diagnosis from three primary dimensions: the lack of efficient fine-tuning strategies, the need for interpretable and reliable models, and the mitigation of computational inefficiencies during edge-end deployment. Subsequently, future prospects addressing these challenges are discussed.

### 4.1. Requirements of DGMs in Rotating Machinery Fault Diagnosis

#### 4.1.1. Fine-Tuning Techniques

DGMs are typically pre-trained on large-scale datasets. They are then fine-tuned on smaller, task-specific datasets. Domain-specific features are learned in this way. The performance gap between the source and target domains is thereby reduced. Fine-tuning has become one of the primary strategies for transfer learning and domain adaptation in DGMs. Two main approaches are commonly used. Full-parameter fine-tuning updates all model weights. Stronger representational capacity is provided, but higher computational and storage overhead are incurred. Parameter-efficient fine-tuning (PEFT) [183] updates only a small subset of parameters. It is more suitable for resource-constrained or multi-task scenarios [184]. PEFT methods can be further classified as follows:

Prefix/Prompt-Tuning [185] achieves adaptation by introducing trainable prefix or prompt vectors.

Adapter-Tuning [186] inserts lightweight adapter modules between model layers and optimizes only their parameters.

Low-Rank Adaptation (LoRA) [187] approximates weight updates through low-rank matrices.

**Figure 8:**
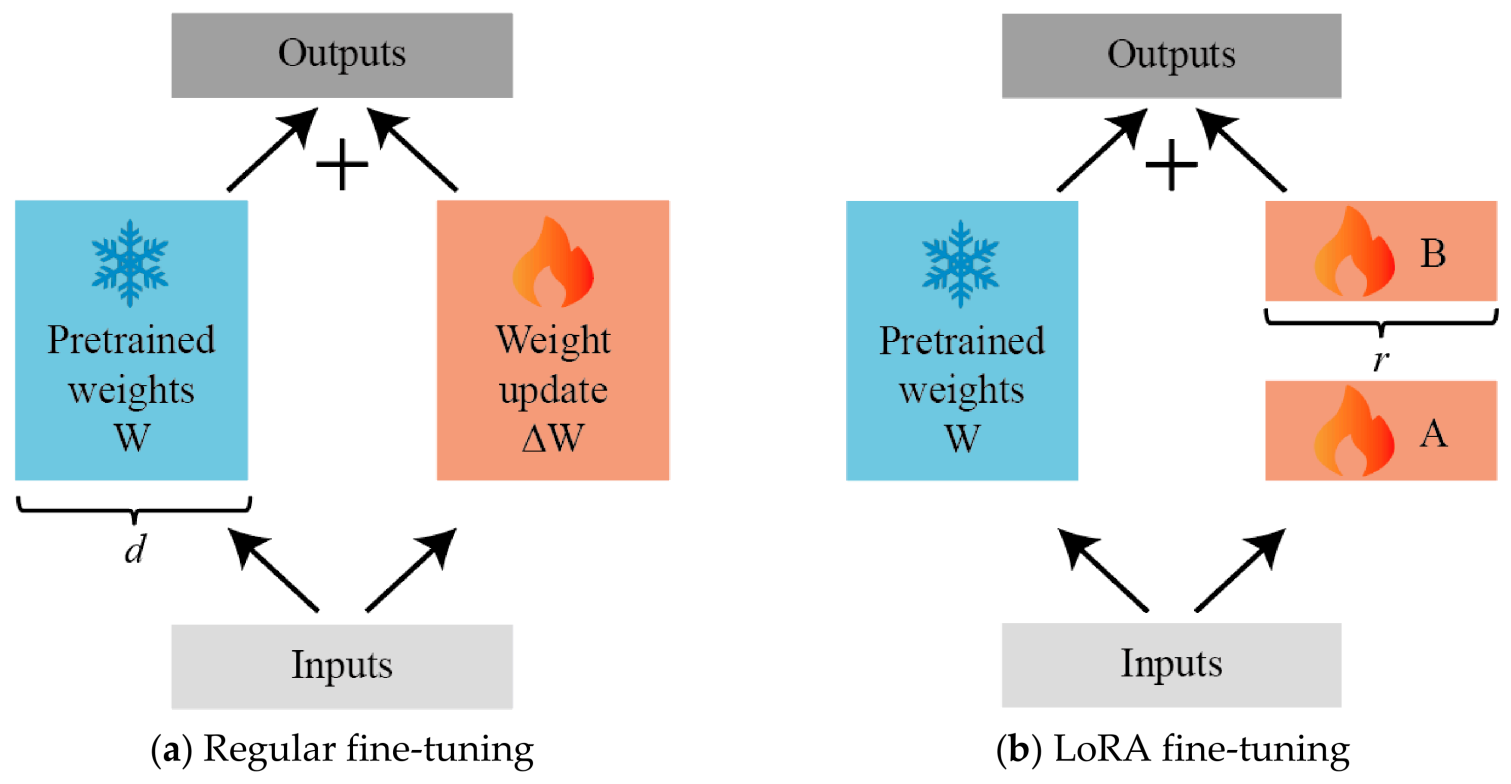
Schematic representation of LoRA fine-tuning paradigms. In contrast to conventional fine-tuning protocols that entail direct modification of pre-trained weight matrices $W\in R^{d\times d}$, LoRA employs a low-rank decomposition strategy to approximate the weight update ∆W. This is achieved by factorizing ∆W into two compact matrices, $A\in R^{d\times r}$ and $B\in R^{r\times d}$, subject to the constraint r≪d, thereby substantially diminishing the cardinality of trainable parameters.

As illustrated in Figure 8, LoRA is applied to decompose parameter updates into low-rank matrices. Adaptation is thus performed with minimal additional inference overhead.

For example, the Hugging Face PEFT library [183] has been used to perform LoRA or adapter fine-tuning on wind turbine gearbox fault data, thereby supporting on-site anomaly detection and condition monitoring.

#### 4.1.2. Interpretability, Comprehensibility, and Reliability

When DGMs are deployed for industrial generation tasks, model interpretability becomes another major obstacle [188]. DGMs combine generative modeling with deep neural networks (DNNs). DNNs are usually regarded as black-box models. Therefore, generated results need to be interpreted, and the internal mechanisms of DGMs need to be elucidated. This field is collectively referred to as eXplainable Generative AI (GenXAI) [189]. Interpretability and comprehensibility are distinct but related concepts. Interpretability is concerned with explaining and justifying model decisions or outputs in human-understandable terms [190]. Comprehensibility emphasizes understanding of the internal processes that govern model behavior [191]. Modern DGMs, especially LLMs, are characterized by large scale. Billions of parameters and large training datasets are typically involved. Significant challenges are thus posed to GenXAI research, compared with traditional DNN models.

Various interpretability techniques have been developed in the GenXAI field. These methods can be primarily categorized into two types: local explanations and global/mechanistic explanations [192]. Local explanations are aimed at interpreting outcomes. Global or mechanistic explanations are intended to explain internal mechanisms.

Traditional DNN interpretability techniques are typically adopted in local methods. Feature attribution methods, such as SHAP, are commonly used [193]. More recent local interpretation techniques leverage LLMs themselves to generate explanations. Post-hoc natural language explanations are constructed by the LLM during its generation process. Data provenance methods, such as Retrieval-Augmented Generation (RAG), are also employed [194].

The second direction involves global or mechanistic explanations. The entire model is explained in this approach. Related techniques have been developed, including internal attribution within DGMs, intra-model explanations, algorithmic understanding, and data influence [195].

**Figure 9:**
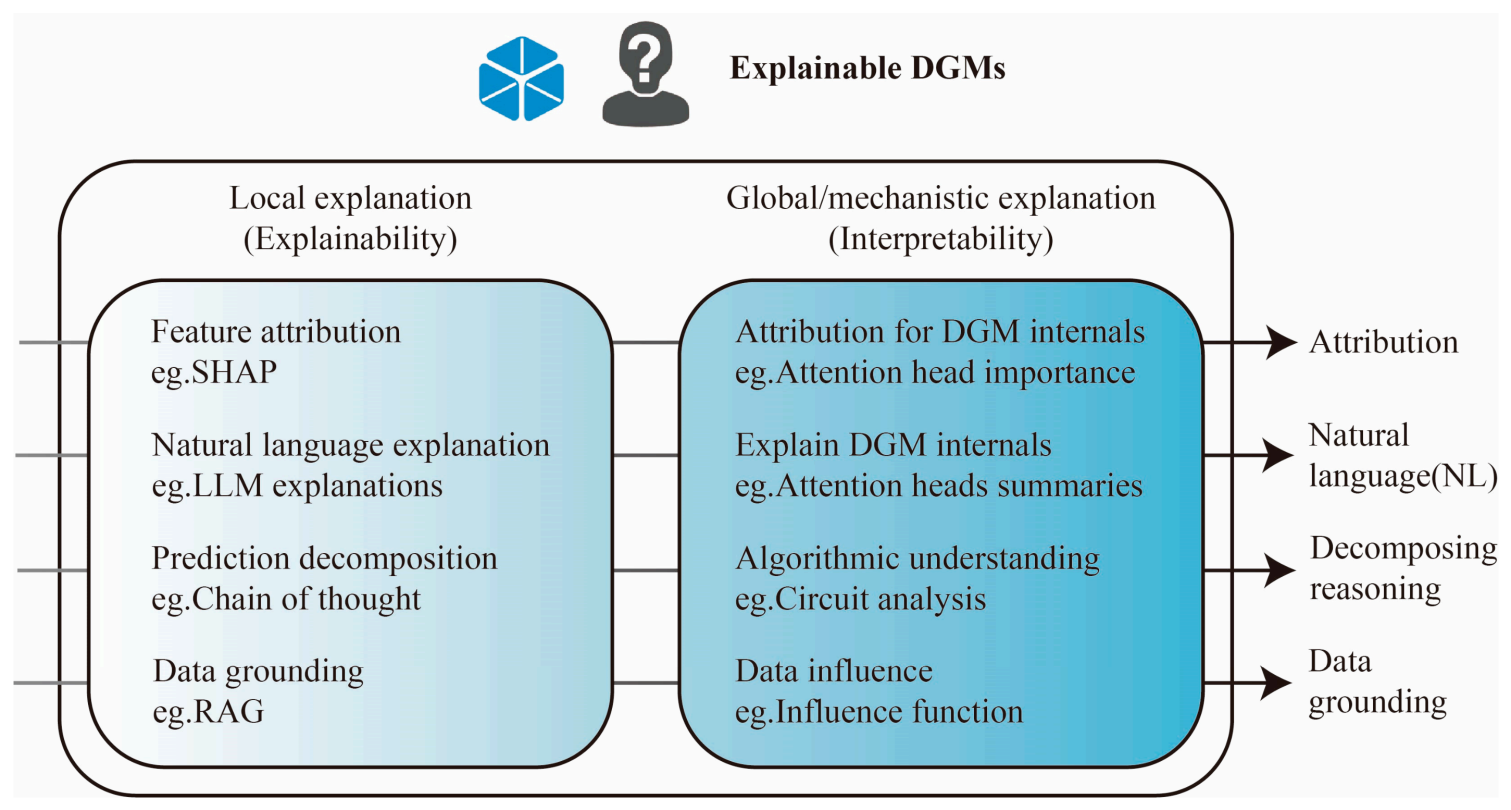
Explainable DGMs are generally divided into two categories: local explanation, which focuses on interpreting the generated content or outputs from the DGM, and global/mechanistic explanation, which is dedicated to interpreting the DGM itself.

The differences between local and global explanations are summarized in Figure 9. Four established perspectives are considered: attribution, natural language, decomposed reasoning, and data provenance.

Trustworthiness remains a critical issue, especially for LLMs [196]. Accurate predictions are generated by leveraging statistical and semantic patterns from training data. However, reliability is inherently constrained by the limitations of DNN models [197]. Grammatical, syntactic, and semantic relationships in language are proficiently mastered by LLMs. Yet it remains uncertain whether words are mapped to real-world entities as humans do. This mapping is crucial for ensuring the trustworthiness and reliability of generated outputs. Hallucinations are prone to occur in current LLMs. Generated content may deviate from facts or user inputs [198]. Barriers to practical implementation may be posed by this discrepancy. The reliability of diagnostic outcomes may also be called into question.

#### 4.1.3. Quantization and Deployment

Deployment aims to transfer algorithms from the development environment to the actual operating environment. Equipment condition monitoring is thereby supported [199]. When large-scale DGMs are deployed on edge devices, model compression strategies should be selected. Model scale, hardware resources, real-time requirements, and accuracy loss are jointly considered in this selection. Memory usage and inference overhead are reduced by model quantization through lowered weight precision. Post-training quantization (PTQ) typically converts models to low precision after fine-tuning is completed. QLoRA performs parameter-efficient fine-tuning on low-precision base models. These two approaches differ in their objectives and applicable stages [200].

For edge nodes with relatively sufficient resources, PEFT-fine-tuned models can be deployed. 8-bit quantization, mixed-precision inference, or cloud-edge collaborative deployment may be adopted. Four-bit quantization becomes more necessary only when GPU memory, power consumption, and computational capacity are strictly constrained.

In addition, network size can be reduced by model pruning and knowledge distillation. Diagnostic performance is largely preserved [201]. A typical deployment pipeline includes:

(1) efficient fine-tuning of DGMs on task-specific datasets;
(2) selection of PTQ, QLoRA, or other compression strategies according to the target hardware;
(3) edge deployment combined with pruning or distillation when necessary.

**Figure 10:**
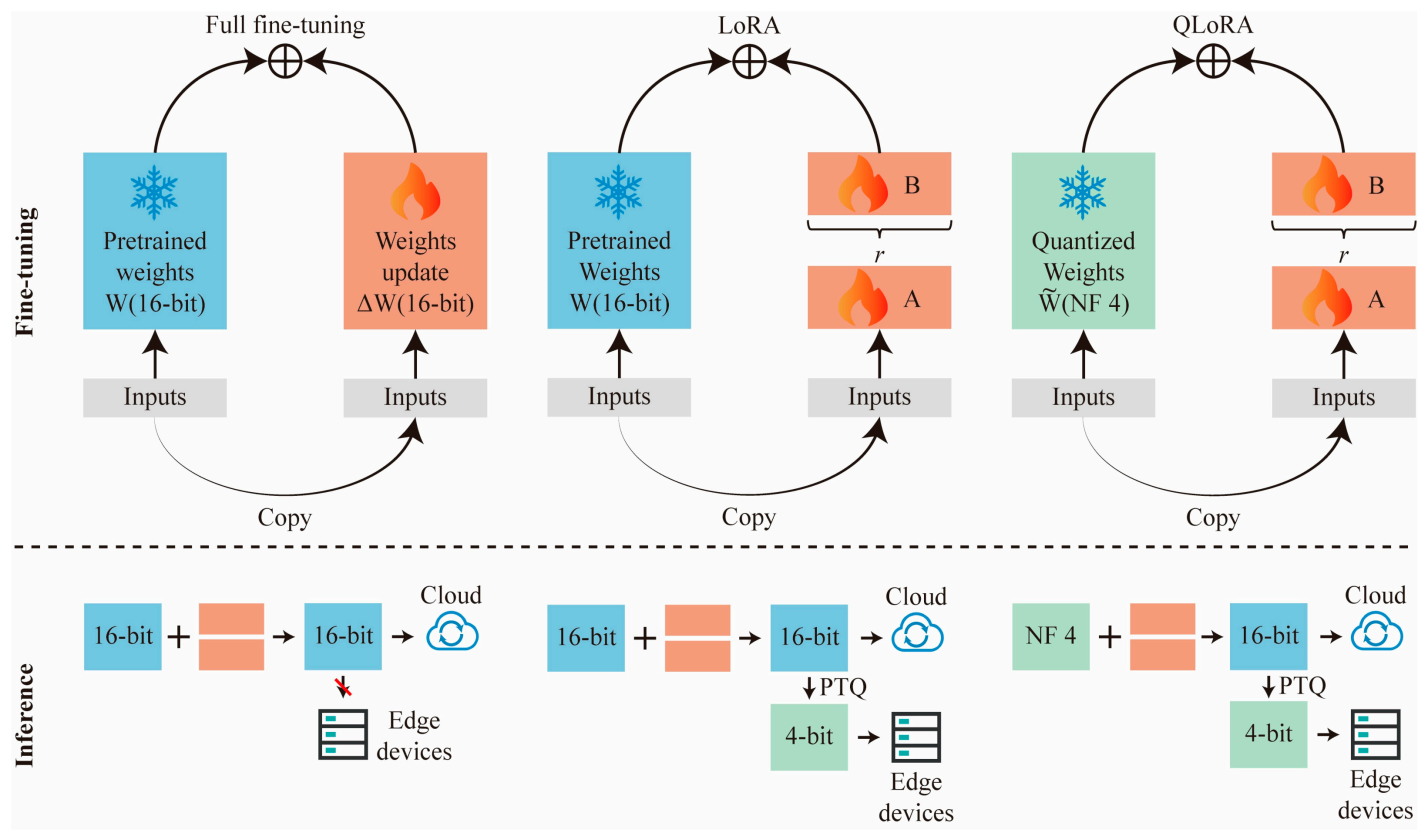
An illustration of DGM quantization and deployment is presented. Fully fine-tuned DGMs are limited to cloud environments. LoRA fine-tuned DGMs are deployable in the cloud with 16-bit floating-point precision. However, quantization to 4-bit integers is required for edge device compatibility. Computational overhead is thereby reduced. QLoRA addresses this challenge. The base pre-trained model is quantized to 4-bit NormalFloat (NF4). Memory consumption is significantly minimized by this data type. Higher accuracy is achieved compared with both 4-bit integers and 4-bit floats.

As shown in Figure 10, LoRA (QLoRA) [202] can serve as a low-resource implementation of LoRA-based fine-tuning for LLMs.

### 4.2. Future Perspectives

#### 4.2.1. Zero-Shot/Few-Shot/Multimodal Data Generation

DGMs, especially LLMs, are gradually transforming the paradigms of few-shot, zero-shot, and multimodal data generation. Robust generalization capabilities are achieved. Few-shot learning aims to achieve high performance with only a minimal number of training samples. Meta-learning techniques are typically integrated [203,204]. New tasks can be rapidly adapted to by the model. Massive training data are not required. Zero-shot learning focuses on diagnosing or predicting unseen samples. It is closely related to the concept of Domain Generalization discussed in Section 3.3.3. DGMs are expected to be pre-trained on large-scale text-sensor paired data. Zero-shot or few-shot reasoning can then be performed for other generation or diagnostic tasks.

Multimodal generation capabilities can be significantly enhanced by DGMs. This enhancement is especially notable when LLMs are leveraged. As discussed in Section 3.3.4, multimodal data fusion is primarily targeted at input data. In contrast, effective generation of multimodal output data can also be achieved by DGMs in this context. Diverse data streams can be generated by DGMs in industrial systems. Examples include vibration spectra, thermal imaging, acoustic emission signals, and operational logs. These data streams can be utilized for rotating machinery fault diagnosis.

#### 4.2.2. Physics-Informed Deep Generative Models

Integrating Physics-Informed Neural Networks (PINNs) [205] with DGMs is poised to become the next prominent research direction in rotating machinery fault diagnosis. The governing equations of controlled systems are embedded as regularization terms or loss functions. The generated data is thereby kept consistent with the physical dynamics observed in the field. Novel opportunities are created when physics-aware principles or rules are combined with LLMs. Non-stationary operating conditions can be simulated in this way. Examples include variable rotational speeds, varying loads, and thermal gradients, which are prevalent in industrial scenarios.

Future research is likely to focus on hybrid architectures, where PINNs guide LLMs to generate physically plausible degradation trajectories (e.g., bearing wear or crack propagation), thereby achieving reliable and timely fault diagnosis. Furthermore, enhancing the physics-aware latent space by directly correlating the generated features with measurable physical parameters (such as force, sound, and temperature) will further improve the interpretability and reliability of the generated data [206,207,208].

#### 4.2.3. Reinforcement Learning Using Deep Generative Models

Reinforcement Learning (RL) has demonstrated remarkable performance in vision-based applications within autonomous driving and robotics. Its application in rotating machinery fault diagnosis is still in its infancy. However, significant attention has been garnered due to its autonomous and adaptive learning characteristics. A pivotal role is also played by RL in training optimization for large-scale models and in solving goal-oriented decision-making problems [209].

Challenges are faced by traditional RL. Sparse rewards, high-dimensional state spaces, and safety-critical constraints are among these challenges [210]. These challenges can be addressed by DGMs. System dynamics are modeled. Environments are simulated. Synthetic fault samples are generated. Latent representations of equipment degradation patterns can be learned by LLMs from historical sensor data. RL agents can then be trained in simulated environments. Rare fault patterns or extreme operating conditions are captured in these environments. Reliance on real-world scenario data is thereby reduced. The robustness of anomaly detection is also enhanced [211,212].

Policy optimization can be achieved by integrating LLMs with RL agents. Future states under different maintenance actions are predicted by LLMs. Policies can thus be explored by agents safely and efficiently. Partial observability issues can be addressed by DGMs. Missing sensor signals are reconstructed. Hidden system states are inferred. RL decision-making capabilities under uncertainty are thereby enhanced. Future research may focus on hierarchical RL frameworks. Long-term maintenance integrated with short-term anomaly detection may be achieved.

## 5. Conclusions

The application scenarios and development trends of Deep Generative Models (DGMs) in rotating machinery fault diagnosis are reviewed in this paper. The challenges of non-stationarity and small sample sizes in rotating machinery fault diagnosis are analyzed, and the limitations of traditional generative models are explored. State-of-the-art DGMs are presented, including Generative Adversarial Networks (GANs), diffusion models, and Large Language Models (LLMs). Furthermore, their applications in this field are categorized into four main domains: data generation, domain adaptation and generalization, multimodal data fusion, and fault diagnosis and anomaly detection (FD & AD). Finally, the existing challenges of DGMs in this field are summarized, and future perspectives are discussed based on current research trends. The main conclusions are drawn as follows:

Although DGMs have demonstrated promising performance in fault diagnosis under dual challenges, their industrial application remains at an early stage, with open issues in fine-tuning, interpretability, and edge deployment. Future work may advance few-shot/zero-shot reasoning and multimodal data generation, leverage physics-informed neural networks (PINNs) to enhance the physical consistency and interpretability of generated data, and integrate DGMs with reinforcement learning to support intelligent operation and maintenance under complex operating conditions. This study is expected to offer useful directions for future research.

## Figures and Tables

**Table 1:** Comparison of DAR, VAE, GAN, diffusion models and large language models.

Models	Basic Structures	Pros	Cons
DAR	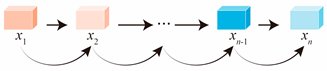	• Aligns with causal logic. • Excels in conditional probability modeling [50].	• Sequential generation lacks backtracking. • Relies on local greedy prediction [51].
VAE	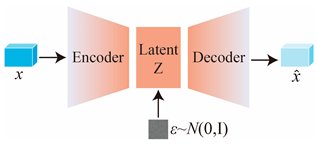	• Incorporates probabilistic regularization [52]. • Grounded in variational inference and ELBO optimization.	• Constrained by MSE-based reconstruction loss. • Based on the assumption of a standard normal prior [53].
GAN	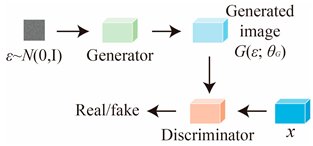	• Leverages adversarial training. • Learns data distribution directly via game theory without explicit density estimation [47].	• The minimax game struggles to reach Nash equilibrium [41]. • Generators tend to generate a single or a few types of samples [54].
Diffusion model	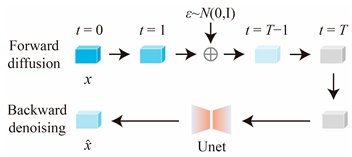	• Achieves comprehensive mode coverage via iterative denoising [46]. • Optimizes a variational bound of likelihood via regression [38].	• Large number of denoising steps [48]. • Hard to control over the generation process.
LLM	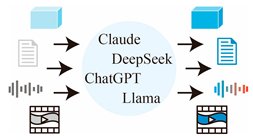	• Pre-trained on massive corpora for cross-domain generalization and prompt-based adaptation. • Exhibits emergent reasoning and parameter-free in-context learning [49].	• Large high-quality training data. • Large computation resources both in the training and inference stages.

**Table 2:** Partial Performance Evaluation Metrics.

Performance Metrics	Mathematical Expressions
Mean Absolute Error (MAE)	$MAE=\frac{1}{N}\sum_{i=1}^{N}\left|x_{i}-{\tilde{x}}_{i}\right|$
Mean Absolute Percentage Error (MAPE)	$MAPE=\frac{1}{N}\sum_{i=1}^{N}\frac{\left|x_{i}-{\tilde{x}}_{i}\right|}{x_{i}}\times 100\%$
Root Mean Square Error (RMSE)	$RMSE=\sqrt{\frac{1}{N}\sum_{i=1}^{N}{\left(x_{i}-{\tilde{x}}_{i}\right)}^{2}}$
Root Mean Square (RMS)	$RMS=\sqrt{\frac{1}{N}\sum_{i=1}^{N}{x_{i}}^{2}}$
Relative Accuracy (ACC)	$ACC=1-\frac{\sqrt{\sum_{i}^{N}{\left(x_{i}-{\tilde{x}}_{i}\right)}^{2}}}{\sqrt{\sum_{i}^{N}{x_{i}}^{2}}}$
Coefficient of determination (R^2^)	$R^{2}=1-\frac{\sum_{i=1}^{N}{\left(x_{i}-{\tilde{x}}_{i}\right)}^{2}}{\sum_{i=1}^{N}{\left(x_{i}-\bar{x}\right)}^{2}}$
Pearson Correlation Coefficient (PCC)	$PCC=\frac{\sum_{i=1}^{N}(x_{i}-\bar{x})({\tilde{x}}_{i}-\bar{\tilde{x}})}{\sqrt{\sum_{i=1}^{N}{\left(x_{i}-\bar{x}\right)}^{2}}\sqrt{\sum_{i=1}^{N}({\tilde{x}}_{i}-\bar{\tilde{x}}{)}^{2}}}$
Maximum Mean Discrepancy (MMD)	${MMD}^{2}=\frac{1}{m^{2}}\sum_{i,j=1}^{m}k(x_{i},x_{j})-\frac{2}{mn}\sum_{i=1}^{m}\sum_{j=1}^{n}k(x_{i},{\tilde{x}}_{j})\;\;+\frac{1}{n^{2}}\sum_{i,j=1}^{n}k({\tilde{x}}_{i},{\tilde{x}}_{j})$
Earth Mover’s Distance (EMD)	${\mathrm{EMD}=\inf}_{\gamma \in \Pi (P,Q)}E_{(x,\tilde{x})∼\gamma }[d(x,\tilde{x})]$
Structural Similarity Index (SSIM)	$SSIM=\frac{\left(2\mu_{x}\mu_{\tilde{x}}+c_{1})(2\sigma_{x\tilde{x}}+c_{2}\right)}{\left(\mu_{x}^{2}+\mu_{\tilde{x}}^{2}+c_{1})(\sigma_{x}^{2}+\sigma_{\tilde{x}}^{2}+c_{2}\right)}$
Crest Factor (CF)	$CF=\frac{{max}_{i}\left|x_{i}\right|}{\sqrt{\frac{1}{N}\sum_{i=1}^{N}{x_{i}}^{2}}}$
Signal-to-Noise Ratio (SNR)	$SNR=20\log_{10}\left(A_{signal}/A_{noise}\right)$

where $x_{i}$ and ${\tilde{x}}_{i}$ denote the i-th sample of the real data and the synthetic data (generated, estimated, or imputed), respectively; N is the number of samples. $\bar{x}=\frac{1}{N}\sum_{i=1}^{N}x_{i}$ and $\bar{\tilde{x}}=\frac{1}{N}\sum_{i=1}^{N}{\tilde{x}}_{i}$ are their mean values. For MMD, m and n are the numbers of real and synthetic samples, respectively, and k(·,·) is a kernel function (a radial basis function kernel is used in this work); for EMD, P and Q are the real and synthetic distributions, Π(P,Q) is the set of joint distributions with marginals P and Q, and d(·,·)  is the ground cost; for SSIM, μ and $\sigma^{2}$ are the local window statistics and $c_{1}$, $c_{2}$ are stabilization constants; for SNR, $A_{signal}$ and $A_{noise}$ are the amplitudes (root-mean-square values) of the signal and noise, respectively, and the amplitude-ratio definition (factor of 20) is adopted.

**Table 3:** Evaluation of Signal Processing, Machine Learning, Deep Neural Networks, and Deep Generative Models Across Different Tasks.

		Signal Processing	Conventional ML/DNNs	DGMs
	Models	Data transformation-based/ Least-squares	SMOTE/CNN/RNN/LSTM	DAR/VAE/GAN/Diffusion
Tasks				
Data augmentation		Refs: Data transformation-based [63,64,65,66]	Refs: SMOTE-based [67,68,69]	Refs: DAR-based [70,71,72,73], VAEs [74,75], GANs [76,77,78,79,80,81,82,83,84,85,86,87,88,89,90,91], Diffusion-based [92,93,94,95,96,97,98,99,100,101,102,103,104]
		Comparison: • [105] DDPM > GAN/AE (MMD score of ≤1.046). • [106] GAN variants (wavelet): (MMD ↓, EMD ↓, SSIM > 0.9). • [107] LSTM/VAE/GAN (ACC ↑). • [108] GAN > SMOTE: (F1 ↑, ACC ↑).		
Data imputation		Refs: Least-squares [109], Bayesian [110]	Refs: KNN [111], PCA [112], RNN [113]	Refs: DAR-based [114,115], VAEs [116], GANs [117,118,119,120], Diffusion-based [121]
		Comparison: • [122] LSTM > RF/LightGBM/MLR: (MAE ↓, RMSE ↓, R^2^ ≥ 0.838). • [123] Bayesian matrix > PCA: (ACC ≥0.95). • [124] Proposed > KNN/RF/MF/RNN: (MAE 7.77, RMSE 10.92). • [125] Proposed > GRU/LSTM: (MAE 0.593, MAPE 0.076, RMSE 0.731). • [126] GAN > SVR/RBF/RNN: (MSE 0.0237, R^2^ 0.9946). • [127] GAN > LSTM/SVR: (ACC ≥ 0.9, Execution time ↓). • [128] Proposed > VAE/Other GANs: (MAE ≤ 4.86, RMSE ≤ 6.1, R^2^ ↑).		
Domain adaptation		Refs: Not applicable	Refs: Gaussian mixture model [129], CNN-based models [130]	Refs: GANs [131,132,133,134,135,136,137,138], Diffusion-based [139]
		Comparison: • [140] DA methods (4 scenarios): (ACC ↑, F1 = 1.0.) • [141] Unsupervised DA: Recall ↑, Prec ↑, ACC ↑, F1 ↑. • [142] Multi-source Diffusion > DNN (ACC ↑).		
Domain generalization		Refs: Not applicable	Refs: Meta-learning techniques [143]	Refs: GANs [91], Diffusion-based model [144]
		Comparison: • [145] Adversarial DG > LSTM/CNN/MS-CNN: (MAE ↓ 0.1117, RMSE ↓ 0.1376). • [146] Meta-DG > DANN/CORAL: (ACC ≥ 83.11). • [147] Meta-representation > Model-Agnostic Meta-Learning (MAML)/Hierarchical Neural Networks: (ACC 15.44% → 31.62%).		

**Table 4:** Evaluation of Signal Processing, Machine Learning, Deep Neural Networks, and Deep Generative Models Across Different Tasks.

		Signal Processing	Conventional ML/DNNs	DGMs
	Models	Kalman filtering/Least-squares	CNN/RNN/LSTM/Transformer	Diffusion/LLM
Tasks				
Multimodal data fusion		Refs: Kalman filtering [156,157,158]	Refs: LSTM-based [159], Transformer [160,161]	Refs: Diffusion-based [162], LLM-based [163,164,165,166,167]
		Comparison: • [168] Multi-scale Transformer > CNN/Single-scale Transformer: (ACC 96.12%). • [169] Swin-Transformer > ResNet18/Efficience V2.		
Fault diagnosis & Anomaly detection		Refs: Not applicable	Refs: CNN-based [170,171,172], Transformer [173,174,175,176]	Refs: Diffusion-based, LLM-based [165,166,167]
		Comparison: • [160] Two-stage encoder Transformer > Extreme Gradient Boosting: (ACC +50%). • [161] Temporal fusion Transformer > Autoregressive Model: (MAE ↑, MSE ↑, MAPE ↑). • [177] Proposed > ShuffleNet/ResNet/DenseNet: (Average ACC 93.1%).		

## Data Availability

No new data were created or analyzed in this study. Data sharing is not applicable to this article.

## Abbreviations

The following abbreviations are used in this manuscript:

DGMs	Deep Generative Models
VAEs	Variational Autoencoders
DARs	Deep Autoregressive Models
DNNs	Deep Neural Networks
DDPMs	Denoising Diffusion Probabilistic Models
LLMs	Large Language Models
GANs	Generative Adversarial Networks
PINNs	Physics-Informed Neural Networks
RL	Reinforcement Learning
PEFT	Parameter-Efficient Fine-Tuning
LoRA	Low-Rank Adaptation
DA	Domain Adaptation
DG	Domain Generalization
FD & AD	Fault Diagnosis and Anomaly Detection
